# How artificial intelligence tools can be used to assess individual patient risk in cardiovascular disease: problems with the current methods

**DOI:** 10.1186/1471-2261-6-20

**Published:** 2006-05-03

**Authors:** Enzo Grossi

**Affiliations:** 1 Medical Department, Bracco SpA Milan, Italy; 2 Centro Diagnostico Italiano, Milan, Italy

## Abstract

**Background:**

In recent years a number of algorithms for cardiovascular risk assessment has been proposed to the medical community. These algorithms consider a number of variables and express their results as the percentage risk of developing a major fatal or non-fatal cardiovascular event in the following 10 to 20 years

**Discussion:**

The author has identified three major pitfalls of these algorithms, linked to the limitation of the classical statistical approach in dealing with this kind of non linear and complex information. The pitfalls are the inability to capture the disease complexity, the inability to capture process dynamics, and the wide confidence interval of individual risk assessment.

Artificial Intelligence tools can provide potential advantage in trying to overcome these limitations. The theoretical background and some application examples related to artificial neural networks and fuzzy logic have been reviewed and discussed.

**Summary:**

The use of predictive algorithms to assess individual absolute risk of cardiovascular future events is currently hampered by methodological and mathematical flaws. The use of newer approaches, such as fuzzy logic and artificial neural networks, linked to artificial intelligence, seems to better address both the challenge of increasing complexity resulting from a correlation between predisposing factors, data on the occurrence of cardiovascular events, and the prediction of future events on an individual level.

## Background

In the past few years a number of algorithms for cardiovascular risk assessment has been proposed to the medical community [1-6]. Their purpose is to assist physicians in defining the risk level of an individual patient with regard to developing major cardiovascular events in the following years.

These algorithms have been drawn from statistical analyses performed on longitudinal study cohorts. These analyses have taken into account events occurring in general populations undergoing adequate follow-up for a sufficient length of time. These algorithms consider a number of variables and express their results as the percentage risk of developing a major fatal or non-fatal cardiovascular event in the following 10 to 20 years. For example, if the algorithm gives origin to a 10% value, it means that 10 out of100 subjects in the reference population at a given time with characteristics similar to those of the subject under evaluation, would develop a cardiovascular event in the following 10 years. These algorithms present some pitfalls linked to the limitations of the classical statistical approach in dealing with this kind of non linear and complex information. The author has identified three. The aim of this paper is to discuss the potential advantage provided by artificial intelligence tools in this specific setting.

## Discussion

### First pitfall: inability to capture disease complexity

The algorithms currently used employ a limited number of variables. This is due to the fact that traditional statistical approaches tend to select only variables which have a high level of linear correlation with the outcome variable.

Classical multivariable statistical techniques are based on a statistical approach, by which only one factor at a time is varied, and the other factors are held constant. With these techniques, a given set of potential predictors with respect to individual patients is difficult to interpret. This is due to the limitations imposed by the underlying non-linear links and the complex interactions between the factors under study.

Artificial neural networks (ANNs) are adaptive models for data analysis particularly suitable for handling nonlinear functions. The Multi-layer perceptron (MLP) is the most widely used type of neural network. It is simple and based on solid mathematical grounds. Input quantities are processed through successive layers of "neurons". There is always an input layer, with a number of neurons equal to the number of variables of the problem, and an output layer, where the perceptron response is made available, with a number of neurons equal to the desired number of quantities computed from the inputs (very often only one). The layers in between are called "hidden" layers in which there are activation functions like logistic sigmoid or hyperbolic tangent. This helps MLP networks to model nonlinear mappings both strongly and mildly. The probabilistic neural networks (PNN) constitute another kind of general classification system, based on the Bayes theory. These networks provide a general solution to pattern classification problems by taking into account the relative likelihood of events and use a priori information to improve prediction. Like MPL, PNN use a supervised training set to develop distribution functions within a pattern layer. These functions, in the recall mode, are used to estimate the likelihood of an input feature vector being part of a learned category, or class. The learned patterns can also be combined, or weighted, with the a priori probability, also called the relative frequency, of each category to determine the most likely class for a given input vector.

ANNs are able to simultaneously handle a very high number of variables notwithstanding the fact that these are not linearly connected. This represents a tremendous advantage in comparison with classical statistical models when the quantity of available information has enormously increased and non linearity dominates. With ANNs one is more concerned about the actual number of variables than about their nature. Due to their particular mathematical infrastructure, ANNs have no limits in handling increasing amounts of variables which constitute the basis for developing recursive algorithms. ANNs can input multiple factor values simultaneously, combining and recombining them in different ways according to specific equations, which are generally non linear. In terms of predictive values and of the number of predictive models, the difference can be explained by the fact that conventional statistics only reveal parameters which are significant for the entire population, whereas artificial neural networks include parameters which might not be significant for the entire population, but which are highly significant on an individual level. Recently, studies on the use of ANNs in the cardiovascular field have been published (table 1). In all of these studies ANNs provided a better predictive accuracy than did traditional statistical techniques. There are two papers in particular that have focused on the prediction of cardiovascular events in conjunction with traditional risk factors in the general population.

**Table 1:** Examples of artificial neural networks analyses in the cardiovascular field.

	Year	No. Pts	Disease	Variables	Results
**Diagnosis**					
Selker	1995	3453	Ischemia	Clinical indicators	ANN superior vs LogR
Ellenius et al	1997	88	MI	Biochemical variables	ANNs give added value
Baxt et al	2002	2204	MI	History, clinical, biochemical EGC	High sensitivity (95%) and specificity (96%)
**Prognosis**					
Baldassarre et al	2004	949	CV event	biochemical, carotid US clinical indicators	ANN superior vs LDA
Voss et al	2002	5159	CV event	Clinical, biochemical indicators	ANN superior vs LogR
Bigi et al	2005	496	Outcome after MI	Clinical, exercise ECG and stress echo	ANN superior vs LDA

MI: Miocardial infarction; CV: cardiovascular; ANN Artificial neural networks; LogR: logistic regression; LDA: linear discriminant analysis

In the Baldassarre study, a database of 949 patients and 54 variables was analyzed to evaluate the capacity of ANNs to recognize patients with a history of vascular events (VE+, n = 196) or without a history of vascular events (VE-, n = 753), on the basis of vascular risk factors (VRFs), carotid ultrasound variables (UVs) or both. The performance of ANNs was assessed by calculating the percentage of correct identifications of VE+ and VE- patients (sensitivity and specificity, respectively) and the predictive accuracy (weighted mean between sensitivity and specificity). Results showed that ANNs can be developed to identify VE+ and VE- subjects more accurately than discriminant analyses. When VRFs and UVs were used as input variables, ANNs provided better predictive capacity, with an accuracy of 80.8% and 79.2%, respectively. The addition of gender, age, weight, height and body mass index (BMI) increased accuracy of prediction to 83.0%. When ANNs were allowed to choose relevant input data automatically (I.S. system-Semeion), 37 out of 54 variables were selected, five of which were UVs. Using this set of variables as input data, the performance of ANNs in their classification task of VE+ patients reached a predictive accuracy of 85.0% and of 92.0%.

In the Voss study, the authors researched to determine whether neural networks improved Logisitic Regression (LR) risk assessment. The authors analyzed data from the Prospective Cardiovascular Munster Study (PROCAM), a large prospective epidemiological study on risk factors of coronary heart disease among working men and women in northern Germany. To estimate the risk of myocardial infarction or death caused by an acute coronary event (coronary events) during a 10 year follow-up among 5159 men aged 35–65 years at recruitment into PROCAM, a multi-layer perceptron (MLP) and probabilistic neural networks (PNN) were employed. Overall, 325 coronary events occurred in this group. The performance of each procedure was assessed by measuring the area under the receiver-operating characteristics curve (AUROC). The AUROC of the MLP was greater than that of the PNN (0.897 versus 0.872), and both exceeded the AUROC by an LR of 0.840. This analysis suggests that using the MLP to identify high-risk individuals candidates for drug treatment would allow prevention of 25% of coronary events in middle-aged men, compared to 15% with LR and 11% with the PNN.

### Second pitfall: inability to capture process dynamics

A major drawback of the probabilistic approach is that prediction tends to behave as a static process. If a given subject has an absolute risk of 62%, it means that there is a 62% probability that he/she will suffer from a major cardiovascular event in the following 10 years, and a 38% probability that he/she will not. The lottery of probability will tell us the truth in the future. In this situation the subject is in a static position in one out of two mutually excluding possibilities: event or non event. There is no possibility to make any sort of inference about a specific risk trend, despite the fact that the same sort of assessment performed 10 years before resulted in an absolute risk of 34%. In fact, even if after 10 years the algorithm shows that there are more probabilities that the subject will suffer from an event, the imposition of binary logic does not allow the mathematical figuring out of a formal and dynamic progression of the risk. The subject will still remain uncertain and apparently his/her fate will still depend on chance. The use of fuzzy logic with artificial intelligence, and the consequent use of a "plausibility" rather than a probability concept, can help overcoming this subtle trap.

Readers might refer to the previous paper to review the difference between probability and plausibility [7] and to a series of papers by Drs. Helgason and Jobe, addressing the issues of complexity and "n" of 1 concept [8-11].

The physician would be paradoxically more precise with fuzzy terminology: he/she could explain to the patient that, given his/her present clinical condition, he/she has reached 62% of the course between a previous safe condition and a future unavoidable event, as a person would explain to one that without noticing it, one is walking from a safe point to the edge of a cliff. This concept introduces a dynamic process. In fact, since in the example the plausibility of an event was 34%, the patient is now told that he/she has very much progressed along a virtual path, and that if nothing will be done to slow this evolution down, there are reasons to think that in another 10 years' time he/she will be very close to a point where an unwanted event will be almost unavoidable (90%). This would make a great difference for the subject, who would not feel part of a cruel lottery anymore, but substantially aware that his/her destiny is already written if nothing will change as to his/her risk factors.

As stated in a previous article [7], in order to deal with a certain degree of uncertainty the use of fuzzy logic would allow the escape from the trap of the probability theory and the advantage of making certain prognoses easier for the patient to understand.

Readers might find an interesting review of the literature regarding medicine and the use of fuzzy logic by Drs. Nieto and Torres [12].

### Third pitfall: wide confidence interval of individual risk assessment

A major unavoidable pitfall of the translation of group statistics onto an individual level is linked to the problem of the wide confidence interval of classifications. Within classical statistical approaches the individual is assimilated into a subgroup of individuals who have, on average, a given probability of an event.

We know that any kind of statistical inference unfortunately is extremely weak in the absence of a "sample", which by definition requires a number >1. For this reason, predictive models can dramatically fail when applied to the single individual.

In a model that has an overall 90% accuracy in predicting an event on a group level, the degree of confidence can drop substantially when applied to a single subject.

Suppose that a predictive model for risk assessment in study data has been developed and validated and that it allows an overall accuracy of 0.9. Suppose that the confidence interval of this predictive rate is 0.06 (0.84–0.96).

The first step is to assess a group of new subjects with our tools. We can reasonably expect to make classification mistakes within a range of 4%–16%. Therefore, 4–16 out of 100 new patients would be incorrectly assessed with regard to their absolute risk.

If a new patient has been classified as in high risk to suffer from myocardial infarction in the next 10 years, the patient might think that there is a 90% chance that he/she has been correctly classified (84% at worst and 96% at best).

Unfortunately the patient's confidence interval in this classification would not be equal to that of the group, since in case of misclassification the patient would suffer from an all or nothing situation (correct prognosis vs incorrect prognosis). This would mean a 100% difference.

In other words, on single subject level the confidence interval would be wider than the mean accuracy rate at a group level.

Is there any solution to this problem?

Since it is not possible to turn a single individual into a group of individuals on which to perform some statistics, one could do the opposite: treat a single individual with a group of statistics. This means using several independent classification models on the same individual. These models make different errors in order to obtain a similar average predictive capacity. Artificial neural networks allow this.

Neural networks can input multiple factor values simultaneously, combining and recombining them in different ways according to specific equations which are generally non linear. Compared with classic statistics in assessing cardiovascular risk [13-15] and in addition to their increased power as modelling techniques, neural networks allow for the building up of a high number of independent models which, have different predictive capacity in classifying patients according to certain targets, due to slight differences in their architecture, topology and learning laws. Overall, neural networks belonging to specific settings do not provide a unique solution, because their performance is determined by several factors, such as the initial randomized incidence of interconnections between nodes, the order of presentation of cases during the training cycle and the number of training cycles. Other variables pertaining to the mathematical attributes of a specific neural network will also affect the final state of a trained neural network, allowing for a very high number of different possible combinations. Evolutionary algorithms have in fact been proposed to find the most suitable design of neural networks, in order to allow a better prediction, given the high number of possible combinations of parameters[16]. In theory, therefore, it is possible to train many different neural networks with the same set of data, with a resulting sizeable assembly of artificial neural networks that have a similar average performance but a different predisposition to make mistakes on an individual level. This way it is possible to produce a large set of neural networks with high training variability able to independently process a set of new patients and to predict their survival plausibility. Up to a thousand answers would be generated for each patient. Therefore when a new patient has to be classified, thanks to this sort of parliament of independent judges acting simultaneously, a specific distribution of output values could be obtained with a resulting descriptive statistics (mean, median, variance, confidence interval, etc.). It is interesting to note that the classification output of neural networks is generally expressed according to the fuzzy logic scheme, along a continuous scale of "degree of membership" to the target class, ranging from 0 (minimum degree of membership) to 1 (maximum degree of membership). According to the above reasoning it could be possible to establish a degree of confidence of a specific classification suitable for the individual patient. It could also be possible to overcome the dogma by which the possibility to make a statistical inference when a sample is composed by just one subject is excluded.

## Summary

The use of predictive algorithms to assess individual absolute risk of future cardiovascular events is currently hampered by methodological and mathematical flaws. The use of newer approaches, such as fuzzy logic and artificial neural networks, linked to artificial intelligence seems to better address both the challenge of the increasing complexity of predisposing factors linked to the occurrence of cardiovascular events data and the prediction of future events on an individual level.

## Competing interests

The author(s) declare that they have no competing interests.

**Figure 1:**
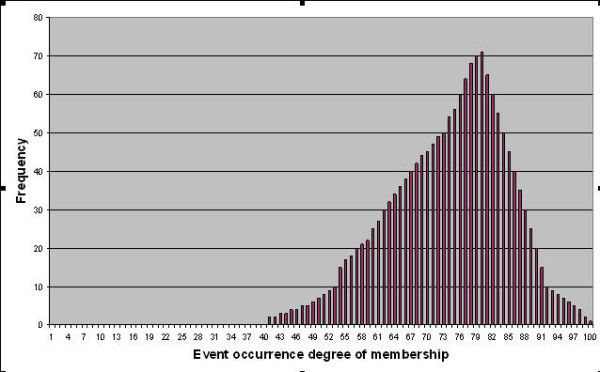
Theoretical distribution of outputs of 1600 different neural networks predicting "plausibility" of event occurrence.

## Pre-publication history

The pre-publication history for this paper can be accessed here:


